# Transcriptome Profile Analysis of Ovarian Tissues from Diploid and Tetraploid Loaches *Misgurnus anguillicaudatus*

**DOI:** 10.3390/ijms160716017

**Published:** 2015-07-14

**Authors:** Weiwei Luo, Chuanshu Liu, Xiaojuan Cao, Songqian Huang, Weimin Wang, Yeke Wang

**Affiliations:** 1 College of Fisheries, Key Lab of Agricultural Animal Genetics, Breeding and Reproduction of Ministry of Education/Key Lab of Freshwater Animal Breeding, Ministry of Agriculture, Huazhong Agricultural University, Wuhan 437000, China; E-Mails: weiweiluo66@163.com (W.L.); chuanshuliu@163.com (C.L.); huangsongqian@163.com (S.H.); wangwm@mail.hzau.edu.cn (W.W.); yekewang@126.com (Y.W.); 2 Freshwater Aquaculture Collaborative Innovation Center of Hubei Province, Wuhan 430070, China

**Keywords:** *Misgurnus anguillicaudatus*, diploid, tetraploid, ovarian tissues, transcriptome

## Abstract

RNA sequencing and short-read assembly was utilized to produce a transcriptome of ovarian tissues from three-year-old diploid and tetraploid loaches (*Misgurnus anguillicaudatus*). A total of 28,369 unigenes were obtained, comprising 10,546 unigenes with length longer than 1000 bp. More than 73% of the unigenes were annotated through sequence comparison with databases. The RNA-seq data revealed that 2253 genes were differentially expressed between diploid and tetraploid loaches, including 1263 up-regulated and 990 down-regulated genes in tetraploid loach. Some differentially expressed genes, such as *vitellogenin* (*Vtg*), *gonadotropin releasing hormone receptor type A* (*GnRHRA*), *steroidogenic acute regulatory protein* (*StAR*), *mitogen-activated protein kinase 14a* (*MAPK14a*), *ATP synthase subunit alpha* (*atp5a*), and *synaptonemal complex protein 1* (*Scp1*), were involved in regulation of cell proliferation, division, gene transcription, ovarian development and energy metabolism, suggesting that these genes were related to egg diameter of the loach. Results of transcriptome profiling here were validated using real time quantitative PCR in ten selected genes. The present study provided insights into the transcriptome profile of ovarian tissues from diploid and tetraploid loaches *Misgurnus anguillicaudatus*, which was made available to the research community for functional genomics, comparative genomics, polyploidy evolution and molecular breeding of this loach and other related species.

## 1. Introduction

Loach (*Misgurnus anguillicaudatus*; Cypriniformes; Cobitidae) is a demersal freshwater teleost, widely distributed in China, Korea, Japan, and other countries in southeastern Asia [1]. The loach can be used as a traditional Chinese medicine or folk remedy for treatments of hepatitis, osteomyeitis, carbuncles, inflammations and cancers, as well as for patient’s recovery from debilities caused by various pathogens and aging [2]. It is also a commercially important species, with a gradually increasing market demand in recent years [3]. Apart from its economic value in East Asia, loach is also a promising model animal to study evolutionary biology for polyploidy. Natural tetraploid loaches with 4*n* = 100 chromosomes appear with sympatric diploid loaches with 2*n* = 50 chromosomes in central China and low frequencies of natural triploid, pentaploid, and hexaploid loach are also found in China [4,5].

Recently, good progress has been made in genome-wide gene expression profiling by the development and application of large scale sequencing techniques, which have served as a powerful and cost-effective tool to obtain gene sequences and develop molecular markers [6]. Combined efforts of high-throughput sequencing and complicated bioinformatics analysis allow access to transcriptome profiling of gonads of several fish, including nile tilapia *Oreochromis niloticus* [7], platyfish *Xiphophorus maculates* [8], olive flounder *Paralichthys olivaceus* [9], rockfish *Sebastiscus marmoratus* [10], rainbow trout *Oncorhynchus mykiss* [11], catfish *Ictalurus punctatus* [12], turbot *Scophthalmus maximus* [13], and striped bass *Morone saxatilis* [14]. Meanwhile, there is almost no report on transcriptome profiling of loach gonad.

Herein, we generated a comparative transcriptome of ovarian tissues from diploid and tetraploid loaches *M. anguillicaudatus* by the application of high-throughput sequencing. The objectives of this study were to enrich genetic resources for *M. anguillicaudatus*, identify and analyze differently expressed genes (DEGs) between diploid and tetraploid *M. anguillicaudatus*, identify some genes related to egg diameters, and obtain information on biological pathways involved in ovarian development, nutrition metabolism and other biological activities as well. Our results will not only provide valuable genomic information for understanding the molecular mechanism of ovarian development of *M. anguillicaudatus*, but also establish a sound foundation for functional genomics, comparative genomics analysis, polyploidy evolution and molecular breeding for this loach and other closely related species.

## 2. Results

### 2.1. Egg Diameters of the Three-Year-Old Mature Loaches of Different Ploidy

**Figure 1:**
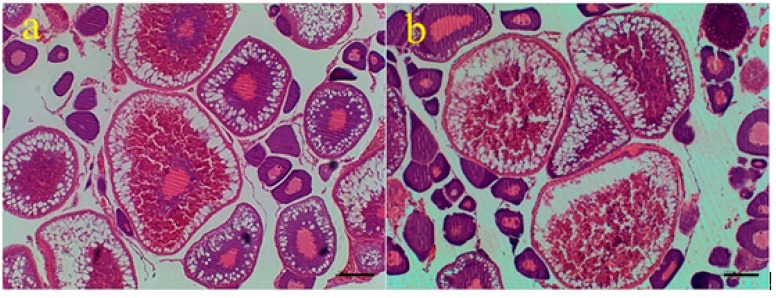
Microstructures of ovarian tissues of diploid (**a**) and tetraploid (**b**) loaches. Note: scale bars =100 µm.

Three-year-old diploid and tetraploid loaches *M. anguillicaudatus* were used in this study. Many mature oocytes were found in ovarian tissues, indicating that these two loaches were of sexual maturity (Figure 1). Diameters of 340 mature eggs from each kind of loach (*i.e.*, diploid and tetraploid) were measured here (Table 1). The average egg diameters for diploid and tetraploid loaches were respectively 1.17 and 1.37 mm. Eggs from tetraploid loaches were significantly larger than those from diploid loaches (*p* < 0.01).

**Table 1:** Diameters of mature eggs from diploid and tetraploid loaches.

Ploidy	Egg Numbers	Egg Diameters (mm)
2*n*	340	1.17 ± 0.04 ^a^
4*n*	340	1.37 ± 0.07 ^b^

Means in the same column with different superscripts were very significantly different (*p* < 0.01).

### 2.2. Sequencing, De Novo Assembly and Functional Annotation

**Figure 2:**
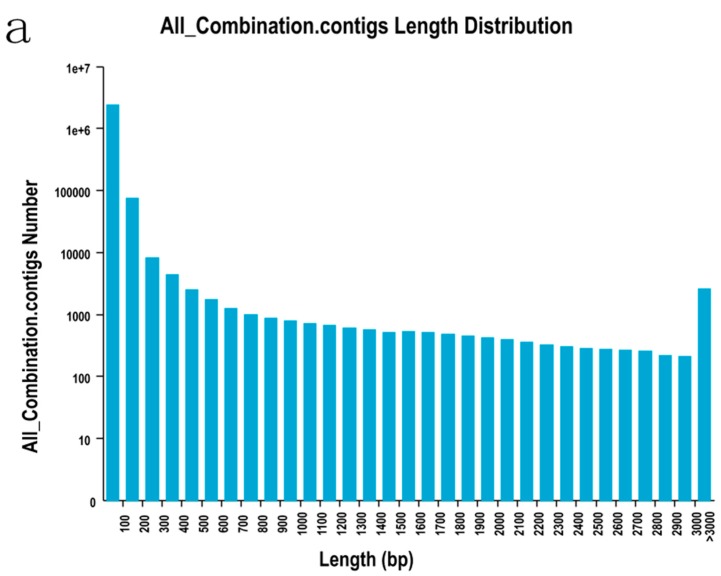

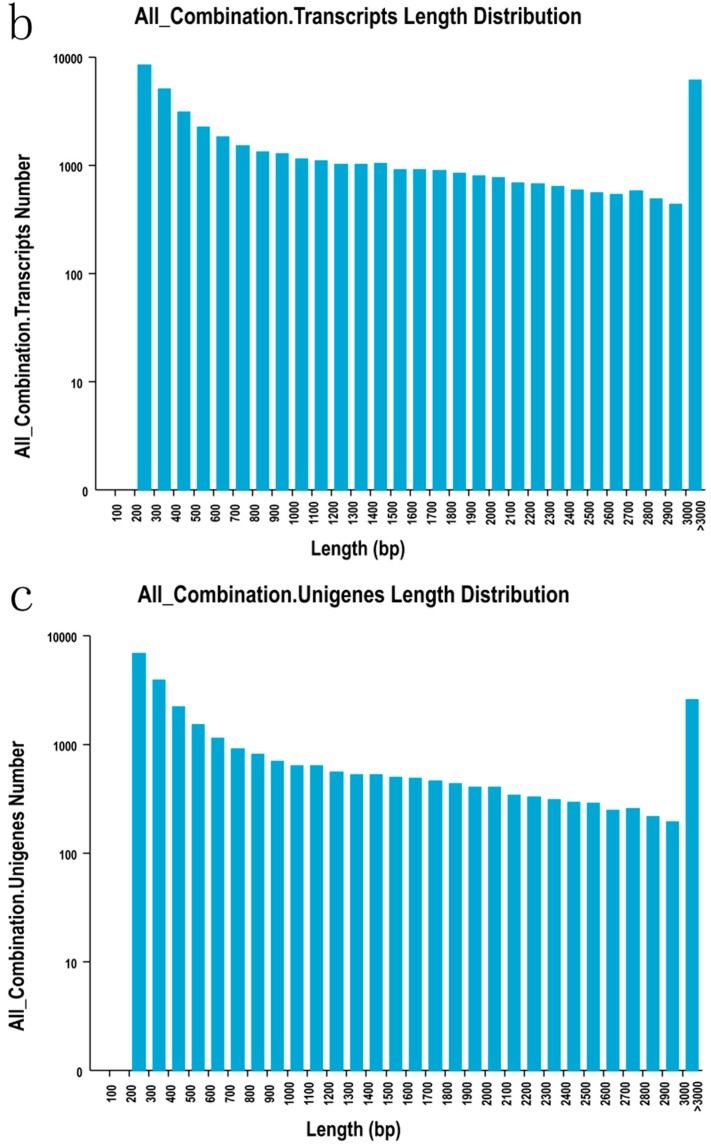
The size distributions of contigs (**a**), transcripts (**b**) and unigenes (**c**).

Illumina-based RNA-Sequencing (RNA-Seq) was conducted with ovarian tissue samples from diploid and tetraploid loaches. A total of 25.42 million of 101 bp paired end reads were generated. After trimming of low-quality reads and short reads sequences, a total of 23.83 million clean reads (93.72%) were obtained (Table 2), and these reads were used for the following analysis. Ovarian tissues of diploid and tetraploid loaches were used to generate 88,454 transcripts and 45,686 unigenes. The N50 values of transcripts and unigenes were 2230 and 1939, respectively. A summary of the assembly data is given in Table 3. The size distributions of contigs, transcripts and unigenes are shown in Figure 2.

**Table 2:** Summary of Illumina expressed short reads production and filtering.

Parameters	Diploid Loach	Tetraploid Loach	In Total
Number of raw reads	12,161,766	13,262,549	25,424,315
Average raw read length (bp)	101	101	101
Number of clean reads	11,416,495	12,411,016	23,827,511
Percentage retained	93.87%	93.58%	93.72%
Average clean read length (bp)	100.99	100.99	100.99

**Table 3:** Summary of the assembly.

Length Range	Contigs	Transcripts	Unigenes
200–300	2,473,738 (99.07%)	8326 (18.04%)	6751 (23.80%)
300–500	6838 (0.27%)	8114 (17.58%)	6034 (21.27%)
500–1000	5600 (0.22%)	8132 (17.62%)	5038 (17.76%)
1000–2000	5433 (0.22%)	9603 (20.81%)	5134 (18.10%)
>2000	5422 (0.22%)	11,977 (25.95%)	5412 (19.08%)
Total number	2,497,031	46,152	28,369
Total length	134,911,717	66,470,417	33,401,504
N50 length	49	2549	2220
Mean length	5403	1440.25	1177.39

**Figure 3:**
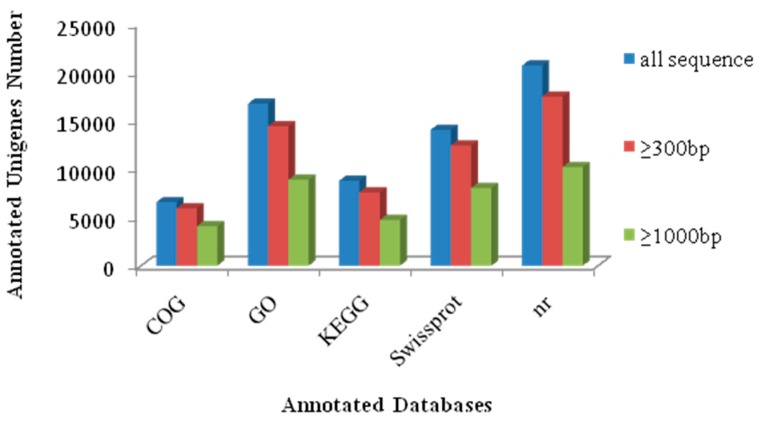
Unigenes annotated by five public databases (COG, GO, KEGG, Swissprot and Nr).

Approximately 73.6% of the unigenes (28,369) were annotated by Blastx and Blastn against five public databases (Nr, Swiss-Prot, KEGG, COG and GO), with a threshold of 10^−5^. Among these unigenes, 20,667, 14,030, 8771, 6562 and 16,722 were identified in the Nr, Swiss-Prot, KEGG, COG and GO databases, respectively (Figure 3).

### 2.3. The Differentially Expressed Genes between the Two Loaches

**Figure 4:**
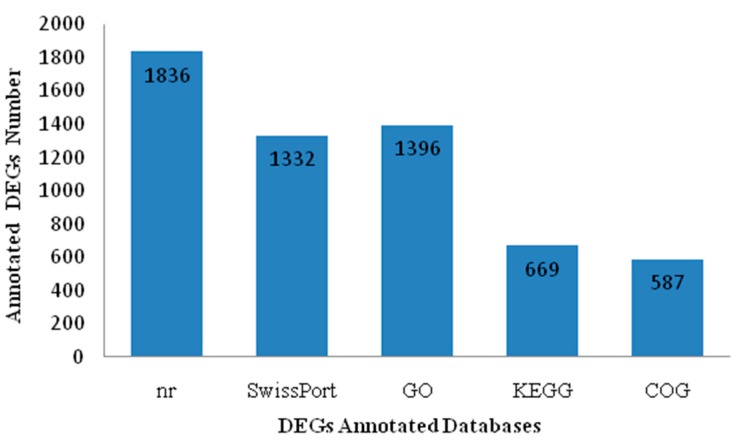
DEGs annotated by five public databases (Nr, SwissPort, GO, KEGG and COG).

A total of 2253 genes were differentially expressed between diploid and tetraploid loaches. 1263 were up-regulated in tetraploid loaches, while 990 were down-regulated, as compared with diploid loaches. Some up-regulated genes, such as *vitellogenin* (*Vtg*), *gonadotropin releasing hormone receptor type A* (*GnRHRA*), *steroidogenic acute regulatory protein* (*StAR*), *mitogen-activated protein kinase 14a* (*MAPK14a*), *ATP synthase subunit alpha* (*atp5a*), *S-phase kinase-associated protein 1* (*Skp1*), *G2/M phase-specific E3 ubiquitin-protein ligase* (*G2E3*), *dynein regulatory complex protein 1* (*drc1*), *cyclin-dependent kinase-like 1* (*CDKL1*) and *synaptonemal complex protein 1* (*Scp1*) (Table 4), were mainly involved in regulation of cell proliferation, division, gene transcription, ovarian development and energy metabolism, indicating these genes might be related to egg diameter of the loach. Moreover, 1836, 1332, 669, 587 and 1396 DEGs were identified in the Nr, Swiss-Prot, KEGG, COG and GO databases, respectively (Figure 4).

**Table 4:** Summary of 10 DEGs related to egg diameter.

#ID	Gene Name	log_2_ Ratio (4*n*/2*n*)	FDR *
comp118882_c0	*Vitellogenin*	4.441525	0.001012
comp20138_c0	*Gonadotropin releasing hormone receptor type A*	4.782572	7.43 × 10^−9^
comp28776_c0	*Steroidogenic acute regulatory protein*	4.097802	0.00883243
comp111884_c0	*Mitogen-activated protein kinase 14a*	5.055045	6.71 × 10^−6^
comp28024_c0	*ATP synthase subunit alpha*	6.593683	7.22 × 10^−15^
comp126960_c0	*S-phase kinase-associated protein 1*	4.516029	0.000597
comp33522_c0	*G2/M phase-specific E3 ubiquitin-protein ligase*	7.034851	0
comp16585_c0	*Dynein regulatory complex protein 1*	8.372893	0
comp32946_c1	*Cyclin-dependent kinase-like 1*	4.097802	0.00883243
comp38857_c0	*Synaptonemal complex protein 1*	11.67117	0

* FDR: false discovery rate, which was used to determine the threshold of *p-*values in multiple tests.

### 2.4. Functional Enrichment Analysis Results for DEGs

**Figure 5:**
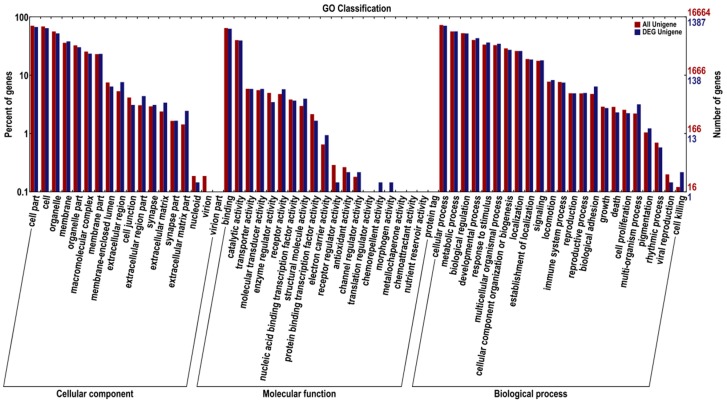
Gene Ontology (GO) classification of the DEGs and all unigenes.

Mapping all DEGs to the terms of the GO database enabled annotation of 1396 DEGs, of which 1133, 1161 and 1244 DEGs could be grouped into the cellular component, molecular function, and biological process categories, respectively (Figure 5). For cellular component, cell part (937, 68.44%), cell (896, 64.18%), organelle (728, 52.15%), membrane (531, 38.04%) and organelle part (421, 30.16%) represented the majorities of this category. Binding (875, 62.68%), catalytic activity (550, 39.40%), transporter activity (81, 5.92%), receptor activity (80, 5.73%) and structural molecule activity (55, 3.94%) represented a high percentage of the molecular function category. In addition, cellular process (986, 70.64%), metabolic process (789, 56.52%), biological regulation (726, 52.01%), developmental process (603, 43.19%) and response to stimulus (508, 36.39%) possessed the majorities of the biological process category.

**Figure 6:**
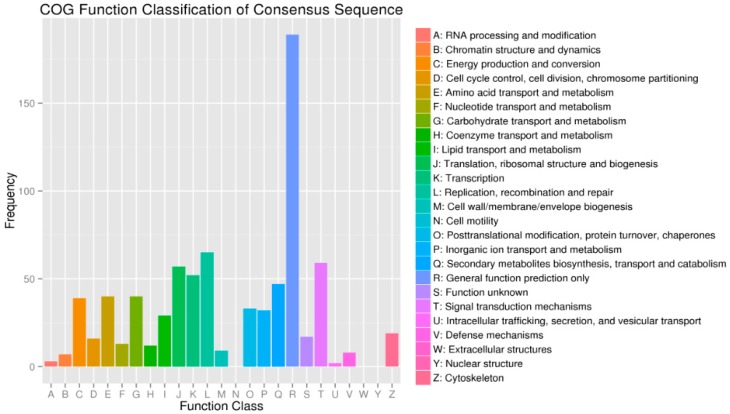
The COG classifications of DEGs.

A total of 587 DEGs were assigned to the COG classifications (Figure 6). Among the 24 COG categories, the top 11 COG categories were as follows: general function prediction represented the largest group (189, 32.20%), followed by replication, recombination and repair (65, 11.07%), signal transduction mechanisms (59, 10.05%), translation, ribosomal structure and biogenesis (57, 9.71%), transcription (52, 8.86%), secondary metabolites biosynthesis, transport and catabolism (47, 8.01%), amino acid transport and metabolism (40, 6.81%), carbohydrate transport and metabolism (40, 6.81%), energy production and conversion (39, 6.64%), posttranslational modification, protein turnover, chaperones (33, 5.62%), inorganic ion transport and metabolism (32, 5.45%) and lipid transport and metabolism (29, 4.94%).

**Figure 7:**
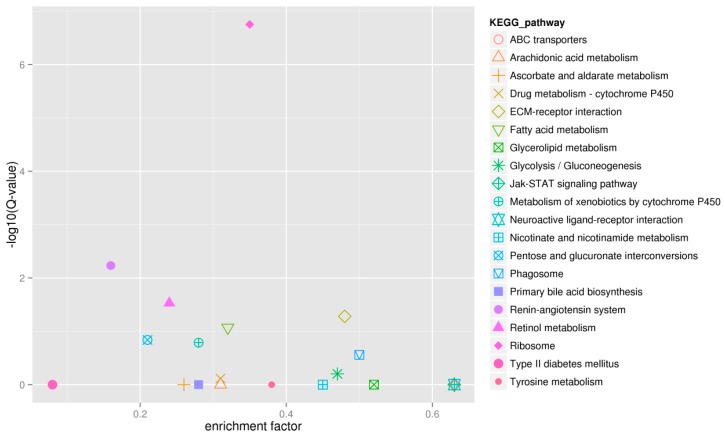
The first twenty enriched pathways.

KEGG pathway annotation enabled us to assign 398 DEGs to 157 pathways. Pathway enrichment analysis identified the first ten enriched pathways including Ribosome (Ko03010), Renin-angiotensin system (Ko04614), Retinol metabolism (Ko00830), ECM-receptor interaction (Ko04512), Fatty acid metabolism (Ko00071), Pentose and glucuronate interconversions (Ko00040), Metabolism of xenobiotics by cytochrome P450 (Ko00980), Phagosome (Ko04145), Glycolysis/Gluconeogenesis (Ko00010) and Drug metabolism-cytochrome P450 (Ko00982). These enriched pathways had functions in cell proliferation, steroidogenesis activity, receptor binding, and energy metabolism, which might convey the differences of ovarian tissues developmental process between diploid and tetraploid loaches. The first twenty enriched pathways are shown in Figure 7.

### 2.5. Validation of Differentially Expressed Genes by Quantitative Real-Time PCR (qPCR)

**Figure 8:**
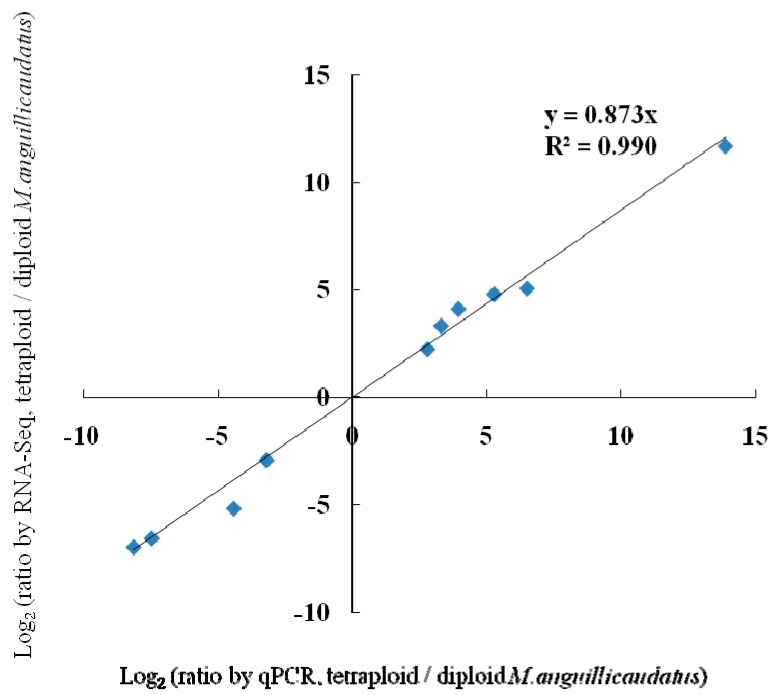
Verification of RNA-Seq by qPCR.

qPCR was performed on 10 selected genes (*GnRHRA*, *CDKL1*, *Creatine kinase B-type CKb*, *adenosylhomocysteinase AHCY*, *Rho guanine nucleotide exchange factor* (*GEF*) *3 ARHGEF3*, *transforming growth factor beta-2 TGF*β*2*, *growth/differentiation factor 7 GDF7*, *Scp1*, *Protein Wnt-11 Wnt11*, and *vitamin D3-25 hydroxylase Cyp27A*) for validating the differentially expressed genes identified by RNA-Seq. Fold changes from qPCR were compared with the RNA-Seq expression profiles (Table 5). The expression patterns revealed by qPCR analysis were similar to those revealed by RNA-Seq for the same genes. In addition, a significantly positive correlation (*r* = 0.996; *p* < 0.01; *n* = 10) was found between the results of RNA-Seq and qPCR for these genes (Figure 8). Thus, RNA-seq could provide reliable data for mRNA differential expression analysis.

**Table 5:** qPCR validation of the randomly selected genes.

Item	Up-Regulated Genes						Down-Regulated Genes			
Gene	*GnRHRA*	*CDKL1*	*CKb*	*AHCY*	*TGF*β*2*	*Scp1*	*ARHGEF3*	*GDF7*	*Wnt11*	*Cyp27A*
RNA-Seq ^a^	4.7826	4.0978	2.2225	5.0550	3.3047	11.6712	−6.5640	−6.9797	−2.9406	−5.1742
qPCR ^b^	5.2700	3.9425	2.7950	6.4900	3.2950	13.8625	−7.4600	−8.1300	−3.1900	−4.4075

^a^ log_2_ Fold Changes of DEGs between diploid and tetraploid loaches by RNA-Seq. Diploid loach was used as control; ^b^ log_2_ Fold Changes of DEGs between diploid and tetraploid loaches by qPCR. β*-actin* was used as reference gene. Gene abbreviations: *Gonadotropin releasing hormone receptor type A* (*GnRHRA*); *Cyclin-dependent kinase-like 1* (*CDKL1*); *Creatine kinase B-type* (*CKb*); *Adenosylhomocysteinase* (*AHCY*); *Transforming growth factor beta-2* (*TGF*β*2*); *Synaptonemal complex protein 1* (*Scp1*); *Rho guanine nucleotide exchange factor* (*GEF*) *3* (*ARHGEF3*); *Growth/differentiation factor 7* (*GDF7*); *Protein Wnt-11* (*Wnt11*) and *Vitamin D3-25 hydroxylase* (*Cyp27A*).

## 3. Discussion

Nowadays, environmental pollution problems are increasingly serious. Some environmental estrogenic substances such as natural and synthetic estrogens, some pesticides, and other manmade chemicals can cause gonadal growth retardation, testicular degeneration, and intersex at relatively low proportions [15,16,17,18]. Therefore, reproductive fitness is a key issue in biology [19]. The ovary is a very important reproductive organ, and ovarian development plays a very important role in reproduction of most animals, including fish. In this study, we used the RNA-Seq technology to present the transcriptome profiling of ovarian tissues from diploid and tetraploid loaches, which are of high economic and medicinal value, and of interest to science as well because of its extensive ploidy variability in nature. The identified DEGs between diploid and tetraploid loaches will not only help us to understand the molecular mechanism of ovarian development and maturation, but also will provide valuable information for studying the phenotypic and functional differences of fish with different types of ploidy. Molecular breeding of loach *M. anguillicaudatus* will also benefit from this study.

Ovarian tissues from diploid and tetraploid loaches used in the present study were both under mature post-vitellogenic conditions. Diameters of the mature eggs demonstrated significant differences between diploid and tetraploid loaches, suggesting that genetic factors most likely accounted for differences of their ovarian development and egg diameters. Within mature ovaries, the oocyte undergoes large increases in size mainly due to the incorporation of vitellogenin [20]. This might require a series of enzymes to provide both hormonal and energy support for synthesis and decomposition of vitellogenin.

We observed that egg diameters of tetraploid loaches were significantly larger than those of diploid loaches. The growing oocyte is considered to be largely transcriptionally inactive, acting as a storehouse of specific maternal RNAs, proteins, and other molecules required for competency for fertilization, initiation of zygotic development, and transition to embryonic gene expression [14]. Of the 2253 DEGs selected with rigorous criteria in the present study, a large proportion of key genes involved in protein processing, fat and energy metabolism, steroidogenic activity, cytoskeleton and cell division were identified. Compared to diploid loach, there were some genes showing marked up-regulation in tetraploid loach, namely *Vtg*, *GnRHRA*, *StAR*, *MAPK14a*, *atp5a*, *Skp1*, *G2E3*, *drc1*, *CDKL1* and *Scp1*, which might account for the difference of egg diameters between diploid and tetraploid loaches. Vtg, the precursor protein for egg yolk, is a large serum phospholipoglycoprotein produced only in the liver of mature females and is transferred by the bloodstream to the ovary. Vtg uptake is stimulated by follicle-stimulating hormone (FSH) [21] and increasing FSH levels are thought to trigger vitellogenesis [22]. In vertebrates, GnRH regulates the synthesis and release of luteinizing hormone (LH) and follicle-stimulating hormone (FSH) from the pituitary gland, thereby regulating steroidogenesis and gametogenesis [23]. The steroidogenic acute regulatory protein (StAR), a member of the StAR-related lipid transfer domain (START) family, is critical to regulated steroidogenesis in vertebrates, controlling oocyte growth (vitellogenesis) and final maturation [24]. MAPK14a, part of the mitogen-activated protein kinase (MAPK) family, is found to act as a crucial mediator for cell differentiation and proliferation [25]. atp5a, one of the energy metabolism enzymes, play a crucial role in steroid hormone and vitellogenin synthesis during ovarian development [26]. Cell cycle phase-specific expression of genes is a principal mechanism controlling cell division, including *Skp1* [27], *G2E3* [28] and *drc1* [29]. CDKL1 is a member of the cyclin-dependent protein kinase (CDK) protein family, a group of serine/threonine kinases [30]. Synaptonemal complex protein 1 (Scp1) is a member of the synaptonemal complex (SC), a meiosis-specific structure essential for synapsis of homologous chromosomes [31]. CDKL1 and Scp1 have also been demonstrated to be important regulators of cell division.

## 4. Materials and Methods

### 4.1. Ethics Statement

All experimental protocols were approved by the Ethics Committee of the College of Fisheries, Huazhong Agricultural University.

### 4.2. Sample Collection and Preparation

The natural loaches were randomly collected from Ezhou city, Hubei province, China. The ploidy status of loach was determined by flow cytometry described as Zhu *et al.* [32]. Then 8 three-year-old mature female loaches (including four diploid and four tetraploid) were chosen. Ages of these loaches were determined by using scale specimens according to Huang *et al.* [33].

**Figure 9:**
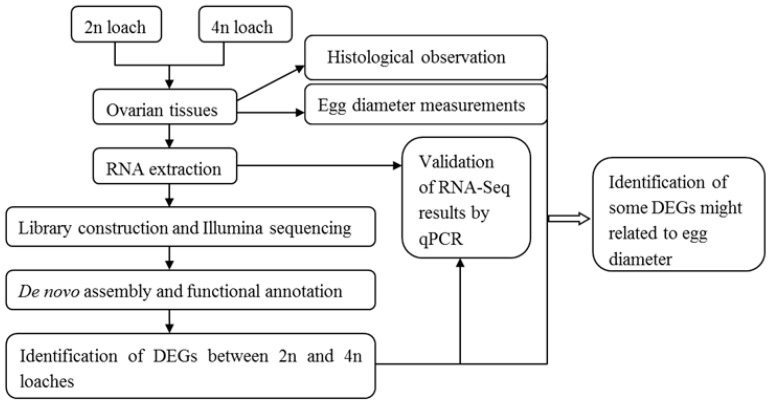
The layout plan of the study design.

For each ploidy (*i.e.*, diploidy and tetraploidy), ovarian tissues were removed from the loaches after anesthetizing with 100 mg/L MS-222. In this experiment, tetraploid sample was used as a treatment group while diploid sample a control group. The ovarian samples of each loach were then divided into three parts. According to Zhao *et al.* [34], the first part was used to measure egg diameters to detect differences in egg-size between the two different ploidies. The second part was fixed in 4% paraformaldehyde solution for the histological observation described as in Cao and Wang [35]. The third part was immediately frozen in liquid nitrogen, and stored at −80 °C, which was used for RNA-Seq and qPCR analysis. Total RNA was extracted from ovarian tissues by using Trizol reagent (Invitrogen, Carlsbad, CA, USA) according to the instructions of the manufacturer. Total RNA concentration and quality were determined by an Agilent bioanalyzer 2100 (Palo Alto, CA, USA). For each ploidy, equal amounts of RNA from the four loaches were pooled to provide templates for the construction of the RNA-Seq library. Figure 9 shows the layout plan of the study design.

### 4.3. Library Construction and Illumina Sequencing

The cDNA library was constructed by using the extracted RNA samples. Poly (A) mRNA was segregated using oligo-dT beads (Qiagen, Dusseldorf, Germany). The fragmentation buffer was added to break all mRNA into short fragments. Random hexamer-primed reverse transcription was used for the first-strand cDNA synthesis. RNase H and DNA polymerase I were used for following generation of the second-strand cDNA. The QIAquick PCR extraction kit was performed to purify the cDNA fragments. These purified cDNA fragments were rinsed by EB buffer for end reparation poly (A) addition and then ligated to sequencing adapters. Thereafter, agarose gel electrophoresis was used for separating the short fragments. The fragments with a size suitable for sequencing criteria were isolated from the gels and enriched by PCR amplification to construct the final cDNA library. The cDNA library was sequenced on the Illumina sequencing platform (Illumina HiSeq™ 2000) using the single-end paired-end technology in a single run, by Biomarker Technologies CO., LTD, Beijing, China. The Illumina GA processing pipeline was used to analyze the image and for base calling.

### 4.4. De Novo Assembly and Functional Annotation

Good quality sequences were necessary for *de novo* assembly analysis. Before assembly, raw sequencing reads were clipped by abandoning adapter sequences and ambiguous nucleotides. Then all clean reads of the two libraries of different ploidy were jointly assembled into contigs implemented by Trinity software. After assembly, contigs longer than 200 bases were used for subsequent analysis. The contigs were connected to obtain the sequence that could not be extended on either end, and the sequence of the unigene was then generated. These unigenes were further spliced and then assembled to acquire maximum length non-redundant unigenes by TGICL clustering software (J. Craig Venter Institute, Rockville, MD, USA). Finally, Blastx with an *E*-value <10^−5^ between the unigenes and the databases non-redundant proteins (Nr), Swiss-Prot, Kyoto Encyclopedia of Genes and Genomes (KEGG) and Clusters of Orthologous Groups (COG) was conducted. GO annotation of these unigenes was produced using Blast2GO based on the results of the NCBI Nr database annotation. Blastn was used for aligning these unigenes to the Nr database, searching proteins with the highest sequence similarity to the given unigenes, accompanied by their protein functional annotations.

### 4.5. Identification of Differentially Expressed Genes (DEGs)

The mapped reads were normalized according to reads per kilobase of exon model per million mapped reads (RPKM) for each unigene between the two pooled samples (*i.e.*, diploid sample and tetraploid sample), which benefited the comparison of unigene expression between samples, and differentially expressed genes (DEGs) identified by the DEGseq package applying the MA-plot-based method with Random Sampling model (MARS) method. DEGs between the two loaches (diploid loaches and tetraploid loaches) were selected with the following filter criteria: FDR (false discovery rate) <0.01 and the absolute value of log_2_ Ratio >1, meaning that the expression difference for each DEG between the two loaches should be at least two-fold. DEGs which might be related to egg diameter were selected with the following criteria: First, based on log_2_ Ratio >2, 810 DEGs were left. Then, we adopted three strategies orderly to excavate genes which might be related to egg diameter from them: (i) delete the DEGs (*n* = 278) whose annotations were not in detail; (ii) egg diameter-related DEGs (*n* = 95) were then obtained; (iii) finally, the top 10 DEGs that showed high-fold changes between the two loaches, related to egg diameter, were selected.

### 4.6. Validation of RNA-Seq Results by qPCR

To examine the reliability of the RNA-Seq results, ten differentially expressed genes (*GnRHRA*, *CDKL1*, *CKb*, *AHCY*, *ARHGEF3*, *TGF*β*2*, *GDF7*, *Scp1*, *Wnt11* and *Cyp27A*) involved in the development of ovarian tissues were selected randomly for validation using quantitative real-time PCR (qPCR). Total RNA was reverse transcribed to first-strand cDNA using reverse transcriptase (Invitrogen). The qPCR was carried out on an iQ5 system (Bio-Rad, Hercules, CA, USA) using SYBR Premix Ex Taq (TaKaRa, Dalian, China), according to the manufacturer’s instructions. The primers for these genes were designed by using Primer Premier 5.0 (Table 6). The reaction mixture (10 µL) comprised 2.5 µL cDNA (1:4 dilution), 5 µL SYBR Premix Ex TaqTM II (TaKaRa), 0.5 µL specific forward primer, 0.5 µL universal primer, and 1.5 µL water. The reactions were performed in an MJ Opticon™-2 machine (Bio-Rad) using the two-step method, with the following parameters: initiation at 95 °C for 30 s followed by 40 cycles of 95 °C for 10 s, 59 °C for 30 s, and 65 °C for 6 s for plate reading. A melting curve was performed from 65 to 95 °C to check the specificity of the amplified product. All experiments were conducted with three biological replicates for each sample. The relative expression levels were normalized to the endogenous control gene β*-actin*, which was expressed stably and equally in ovarian tissues of diploid and tetraploid loaches, and expression ratios were calculated by using the 2^−^^ΔΔ*C*t^ method.

**Table 6:** Primers used for quantitative real-time PCR (qPCR) verification.

Gene-ID	Nr_annotation	Forward Primer (5′–3′)	Reverse Primer (5′–3′)	Product Size (bp)
comp20138_c0	*GnRHRA*	CCAGCCAGAGATGTTGAAGGT	GCGGAAGGAAGGAGTGTAAAGA	118
comp32946_c0	*CDKL1*	AGTCTGCGAGAGGAAAGCGA	GCAAATACCCAGGTGGAAGG	94
comp42021_c0	*CKb*	AGCCCTGTATGAGAAGTTGCG	TATCCTCCATGCCTGTCCCTA	192
comp34136_c1	*AHCY*	GATACGGAGAGGTGGGAAAAGG	TGTAGAGCACAAATGGGGTCAA	94
comp22497_c0	*ARHGEF3*	CTCAAGACTGGAGAGGCGGA	GGTCGGGTCAAAACCAACACTA	182
comp34529_c0	*TGF*β*2*	GGAGAAGAACGCCTCAAACCT	CGCACCACCTTACTGTCAATG	162
comp35042_c1	*GDF7*	TTCAAAGAACTGGGTTGGGATG	AGCGTCTGGATGATGGCGT	131
comp38857_c0	*Scp1*	TCTCCGCCAGTTCAGGGTTA	CTCTGGACTGCCACATTTTGATT	217
comp38576_c0	*Wnt11*	CCAGAGCACCTTCAGCGACAT	CAGCCACCCCATCTAAAATCC	231
comp29375_c1	*Cyp27A*	GAGACCGTCCTGTTTTTCCCA	GATACATCCCCTCCACCTGCT	169

## 5. Conclusions

This is the first report of the ovarian transcriptome of diploid and tetraploid loach *M. anguillicaudatus* obtained by using RNA-seq. We generated a large number of ESTs and identified numerous differentially expressed genes of ovarian tissue between these two loaches. According to DEGs annotation information, we found some genes related to egg diameters of the loach. Gene products do not act independently but rather influence biological processes through networks of interactions. Further functional characterization of these genes by using transgenic, over expression, knockout and knockdown strategies may help elaborate the molecular mechanisms controlling egg sizes among different ploidy fish. Our results also provide the reference values for functional genomics, comparative genomics analysis, polyploidy evolution and molecular breeding for this loach and other closely related species.
